# The Pro Allele of the p53 Codon 72 Polymorphism Is Associated with Decreased Intratumoral Expression of BAX and p21, and Increased Breast Cancer Risk

**DOI:** 10.1371/journal.pone.0047325

**Published:** 2012-10-10

**Authors:** Katharina Proestling, Alexandra Hebar, Nina Pruckner, Erika Marton, Ursula Vinatzer, Martin Schreiber

**Affiliations:** Department of Obstetrics and Gynecology, Medical University of Vienna, Vienna, Austria; University of Texas MD Anderson Cancer Center, United States of America

## Abstract

**Background:**

The TP53 Arg72Pro polymorphism encodes two p53 variants with different biochemical properties. Here we investigated the impact of this polymorphism on the expression of key p53 target genes in a panel of human breast carcinomas, breast cancer risk, and age at onset.

**Methodology/Principal Findings:**

The Arg72Pro polymorphism was genotyped in 270 breast cancer patients and 221 control subjects. In addition, the Arg72Pro genotype of 116 breast tumors was determined, and correlated with intratumoral mRNA expression of TP53 and its key target genes MDM2, p21, BAX, and PERP, as quantified by qRT-PCR. We found a significantly increased breast cancer risk associated with the Pro-allele (per-allele odds ratio, 1.46; 95% confidence interval, 1.08–1.99), and a significantly later mean age at breast cancer onset for Pro/Pro patients (63.2±18 years) compared to Arg/Arg patients (58.2±15 years). The frequency of somatic TP53 inactivation was 25.4% in Arg/Arg, 20.9% in Arg/Pro, and 16.7% in Pro/Pro patients, which may reflect a higher selective pressure to mutate the Arg-allele. The median mRNA levels of p21 and BAX in the tumors of Pro-allele carriers were significantly reduced to 55.7% and 76.9% compared to Arg/Arg patients, whereas p53, MDM2 and PERP expression were hardly altered.

**Conclusions/Significance:**

The p53^72Arg^ variant appears to be a more potent *in vivo* transcription factor and tumor suppressor in human breast cancer than the p53^72Pro^ variant. The Arg72Pro genotype has no significant effects in patients with TP53 mutated tumors, in which p53 is non-functional.

## Introduction

The tumor suppressor protein p53, encoded by the TP53 gene, is a transcription factor which is activated by a diverse range of cellular stresses. Once activated, p53 induces or represses hundreds of target genes with a key role in cell cycle arrest, apoptosis, senescence, and DNA repair, thus preventing tumor development and progression [1]. A key target gene executing p53’s role in cell cycle arrest is the CDK inhibitor p21, which is induced by p53 by direct binding to the p21 promoter [2]. p21 binds tightly to complexes of cyclins and cyclin-dependent kinases (CDKs), inhibiting their function. Accordingly, induction of p21 arrests the cell cycle in the G1 phase, thus mediating the function of p53 in preventing the division of DNA-damaged cells [1], [3], [4]. In addition, p21 may also be induced by a p53-independent pathway [5]. Interestingly, mice lacking p21 do not exhibit an increased cancer incidence, in contrast to mice lacking p53 [6]. This and additional evidence suggest that the apoptotic program plays an essential role in p53 mediated tumor suppression. Activated p53 induces apoptosis primarily via activation of target genes such as BAX (a pro-apoptotic member of the BCL2 family that induces cell death by acting on mitochondria), BBC3 (Puma), PMAIP1(Noxa), and APAF1 [1]. Another p53 target gene with a role in apoptosis is PERP, a member of the PMP-22/gas3 family [7], [8]. PERP expression is reduced in many human breast cancer cell lines compared with untransformed cells, and PERP deficiency promotes the development of mammary tumors in mice [9]. A further key target gene of p53 is MDM2, which acts as a p53 specific ubiquitin ligase and thus targets p53 for proteasomal degradation. In addition, MDM2 directly blocks the transcriptional activity of p53 and stimulates its nuclear export [10]–[13]. Thus, MDM2 is both a target gene and a major negative regulator of p53, forming an auto-regulatory feedback loop which prevents activation of p53 in the absence of stress stimuli [1], [14], [15].

Somatic inactivation of TP53 by mutation is the most common genetic alteration in human cancer, and often results in functionally compromised p53 unable to efficiently induce transcription and suppress tumorigenesis [16], [17]. In breast cancer, TP53 is found mutated in approximately 20–40% of all cases [18]–[20]. Besides mutations, genetic polymorphisms in TP53 could also affect some of its functions [21]–[27]. A common single nucleotide polymorphism (SNP) is rs1042522, which is located in the proline-rich region in exon 4 of TP53. This polymorphism, hereafter referred to as Arg72Pro, encodes either an arginine (R; codon CGC) or a proline (P; codon CCC) residue as amino acid 72. Importantly, these two p53 variants exhibit different biochemical properties [27]. p53^72Arg^ is a more efficient inducer of apoptosis than p53^72Pro^, and thus may increase the responsiveness to chemotherapy [21], [23], [26]. Conversely, p53^72Pro^ has been reported to be a more efficient activator of DNA-repair and cell cycle arrest than p53^72Arg^ [22], [23], [27]. p53 being a transcription factor, these biological differences are likely due to differential transcriptional activities of the two codon 72 variants. Unfortunately, analyses of the relative potencies as a transcription factor of p53^72Pro^ and p53^72Arg^ have been done almost exclusively in vitro, and have produced partly contradictory results. In transient transfections of p53 null mouse fibroblasts, p53^72Pro^ activated reporter constructs to ∼2-fold higher levels than p53^72Arg^ [27]. Reporter assays in H1299 human p53 null cells revealed that activation of p53^72Pro^ induced p53 target genes with a key role in DNA repair more efficiently than p53^72Arg^, such as GADD45, p53R2 and p48 [23]. On the other hand, p53^72Pro^ was less efficient in inducing p21 and MDM2 in this study, although the latter differences were not significant [23]. In contrast, the mRNA of the pro-apoptotic p53 target genes PUMA, PIGPC1, and AIP1 was induced more efficiently by p53^72Arg^ in another *in vitro* study with H1299 cells, whereas no difference was observed for p21 or MDM2 [26]. In an *in vitro* analysis of 34 p53 target genes in Saos-2 osteosarcoma cells, several genes were induced more efficiently by p53^72Arg^ than p53^72Pro^, particularly those with a role in apoptosis; the largest difference (∼80-fold) was observed for the PERP gene [8]. This study supports the conclusion that p53^72Arg^ is better at stimulating apoptosis than p53^72Pro^, whereas there was no evidence that p53^72Pro^ is better at stimulating any p53 function [8]. Finally, *in vivo,* p21 expression was lower in the peripheral leukocytes of Pro-allele carriers [25].

The TP53 Arg72Pro SNP is a biologically plausible candidate low penetrance genetic risk factor, and its association with breast cancer risk has been investigated by several studies. In some studies the Pro-allele has been associated with increased breast cancer risk [28], [29]. Other studies found the Arg/Arg genotype associated with breast cancer predisposition [30]–[32]. Yet other studies, including most of the newer and larger studies and meta-analyses, did not detect any association of the Arg72Pro polymorphism with breast cancer risk [33]–[41]. These discrepancies have been suggested to be due to the failure to determine the mutational status of p53 in the study populations and/or the observed latitudinal differences in allele frequency [42]. Pro is the ancestral allele (∼95% allele frequency in Africans), and the frequency of the Arg allele progressively increased as populations migrated further North. For example, an Arg allele frequency of ∼80% was observed in Northern Europe [43]. Breast cancer patients with the Pro/Pro genotype had a significantly poorer survival than Arg-carriers [35], [44]. Moreover, breast cancer patients with the Pro/Pro genotype exhibited poorer response rates after receiving anthracycline-based chemotherapy [45]. Furthermore, the Pro/Pro genotype was found to be overrepresented in lobular and in grade 1 breast tumors [35]. Here we have evaluated the association of the TP53 Arg72Pro polymorphism with breast cancer risk, age of onset, and clinical characteristics in a hospital-based case-control study of 267 consecutive breast cancer patients and 220 controls. In addition, the Arg72Pro SNP was genotyped in 116 fresh frozen breast tumor tissue samples. Quantitative analysis of mRNA levels in these tumor tissues revealed that the Pro allele is associated with significantly reduced levels of p21 and BAX expression. Consistent with its apparently weaker in vivo transcriptional capacity, the Pro-allele was associated with an increased breast cancer risk. The data thus highlight the critical impact of the Arg72Pro SNP on breast cancer biology.

**Table 1:** Clinical characteristics of the study population, and frequency of the TP53 Arg72Pro genotypes in the indicated subpopulations.

	Total		Arg/Arg	Arg/Pro	Pro/Pro
All subjects	487		250 (51.3%)	210 (43.1%)	27 (5.5%)
Patients	267		125 (46.8%)	123 (46.1%)	19 (7.1%)
Controls	220		125 (56.8%)	87 (39.5%)	8 (3.6%)
Patient subgroups					
Mean age	58.7±14.3		58.2±15.0	58.5±13.0	63.2±18.0
Median age	60.2		60.2	58.8	67.6
Menopausal	pre	61	30 (49.2%)	25 (41.0%)	6 (9.8%)
status	post	172	78 (45.3%)	83 (48.3%)	11 (6.4%)
	na	34	17 (50.0%)	15 (44.1%)	2 (5.9%)
Tumor size	pT1	132	64 (48.5%)	57 (43.2%)	11 (8.3%)
	pT2	56	19 (33.9%)	33 (58.9%)	4 (7.1%)
	pT3, pT4	11	6 (54.5%)	4 (36.4%)	1 (9.1%)
	other, na	68	36 (52.9%)	29 (42.6%)	3 (4.4%)
Tumor type	ductal	148	74 (50.0%)	65 (43.9%)	9 (6.1%)
	lobular	47	18 (38.3%)	24 (51.1%)	5 (10.6%)
	other, na	72	33 (45.8%)	34 (47.2%)	5 (6.9%)
Stage	0, I	112	54 (48.2%)	49 (43.8%)	9 (8.0%)
	II	63	25 (39.7%)	35 (55.6%)	3 (4.8%)
	III, IV	20	11 (55.0%)	7 (35.0%)	2 (10.0%)
	na	72	35 (48.6%)	32 (44.4%)	5 (6.9%)
Grade	pG1	43	19 (44.2%)	23 (53.5%)	1 (2.3%)
	pG2	114	47 (41.2%)	55 (48.2%)	12 (10.5%)
	pG3	88	49 (55.7%)	34 (38.6%)	5 (5.7%)
	na	22	10 (45.5%)	11 (50.0%)	1 (4.5%)
Nodal status	pN0	143	68 (47.6%)	65 (45.5%)	10 (7.0%)
	pN+	53	23 (43.4%)	27 (50.9%)	3 (5.7%)
	na	71	34 (47.9%)	31 (43.7%)	6 (8.5%)
p53 status	pos	56	29 (51.8%)	24 (42.9%)	3 (5.4%)
	neg	191	85 (44.5%)	91 (47.6%)	15 (7.9%)
	na	20	11 (55.0%)	8 (40.0%)	1 (5.0%)
ER status	pos	196	86 (43.9%)	95 (48.5%)	15 (7.7%)
	neg	58	30 (51.7%)	24 (41.4%)	4 (6.9%)
	na	13	9 (69.2%)	4 (30.8%)	0 (0.0%)
PR status	pos	137	56 (40.9%)	70 (51.1%)	11 (8.0%)
	neg	117	60 (51.3%)	49 (41.9%)	8 (6.8%)
	na	13	9 (69.2%)	4 (30.8%)	0 (0.0%)
HER2 status	pos	51	30 (58.8%)	16 (31.4%)	5 (9.8%)
	neg	200	85 (42.5%)	101 (50.5%)	14 (7.0%)
	na	16	10 (62.5%)	6 (37.5%)	0 (0.0%)

Numbers of patients in each of the indicated subgroups are shown. Numbers in parentheses indicate the fraction of patients (%) in each row with genotypes Arg/Arg, Arg/Pro and Pro/Pro, respectively. na, status not available; ER, estrogen receptor; PR, progesterone receptor. p53 IHC positivity indicates a TP53 mutation [53]. All p-values of subgroup comparisons were >0.05 (Chi^2^ tests).

**Figure 1:**
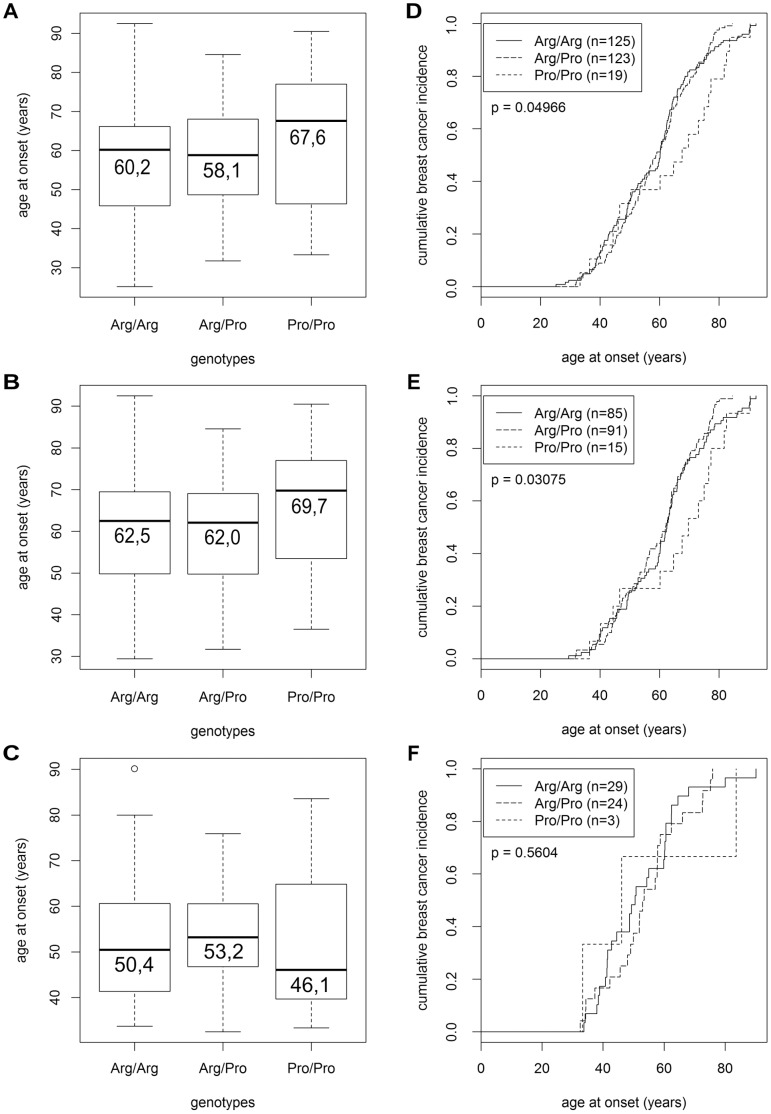
Age at breast cancer onset by Arg72Pro genotypes. Boxplots (**A–C**) and curves of the cumulative breast cancer incidence at the indicated age at onset (**D–F**) are shown for unselected patients (**A, D**), patients with p53 negative tumors (**B, E**), and patients with p53 positive tumors (**C, F**). The genotypes of these patients are indicated in each panel, their numbers in panel **D–F**, and their median age at onset in panel **A–C**.

## Materials and Methods

### Study Population

This study was approved and is annually reviewed by the Institutional Review Board (“Ethikkommission”) of the Medical University of Vienna, Austria (MUV). Blood samples from 270 consecutive breast cancer patients treated between 2002 and 2004 at the Department of Obstetrics and Gynecology, MUV, were collected. Patients with benign gynecological lesions and healthy donors without any malignancies in their personal history were defined as controls in this study (n = 221). Like the patients, controls were enrolled between 2002 and 2004 at the Department of Obstetrics and Gynecology, MUV, and written informed consent was obtained from all participants. Fresh-frozen tumor tissue from 118 breast cancer patients was collected between 1991 and 1994. Only women of Caucasian background from the same geographical area were included as patients or controls. For technical reasons, the genotype could not be determined for four blood samples (3 patients and 1 control) and for two tumor tissue samples. Accordingly, all further analyses were based on the 487 blood samples and 116 tumor tissue samples whose genotype could be ascertained. Clinical and histopathological characteristics of the study populations are provided in Table 1 for the “blood cohort” and Table S1 for the “tumor tissue cohort”.

**Table 2:** Odds ratios and 95% confidence intervals for TP53 Arg72Pro genotypes or alleles and breast cancer risk.

	unadjusted			adjusted*		
Genotypes/Alleles	OR	95% CI	p-value	OR	95% CI	p-value
Pro/Pro vs. Arg/Arg	2.38	1.01–5.93	0.046	3.06	0.74–12.67	0.111
Pro/Pro vs. Arg/Pro	1.68	0.71–4.23	0.247	2.02	0.47–8.66	0.325
Arg/Pro vs. Arg/Arg	1.41	0.98–2.05	0.067	1.53	0.91–2.58	0.113
Pro/Pro + Arg/Pro vs.Arg/Arg	1.49	1.04–2.14	0.028	1.63	0.98–2.71	0.060
Pro/Pro vs. Arg/Pro +Arg/Arg	2.03	0.87–4.73	0.089	2.55	0.63–10.37	0.164
Pro vs. Arg	1.46	1.08–1.99	0.013	1.60	1.02–2.50	0.036

Analyses of breast cancer cases vs. controls of the indicated genotypes or Pro vs. Arg alleles are shown. 95% CI, 95% confidence intervals. *adjusted for age and menopausal status.

### DNA Isolation and Analyses

Genomic DNA was extracted from patients’ peripheral lymphocytes or 118 fresh-frozen primary tumor tissues with a QIAamp DNA Blood Midi kit (Qiagen, Hilden, Germany), or High Pure PCR Template Preparation Kit (Roche, Vienna, Austria), respectively, following the manufacturers’ protocols. Genotyping of SNP rs1042522 (TP53 Arg72Pro) in blood samples was performed by polymerase chain reaction and restriction fragment length polymorphism assay (PCR-RFLP) as described [32]. Tumor samples were genotyped by sequencing using the ABI PRISM BigDye Terminator v3.1 Cycle Sequencing Kit (Applied Biosystems, Vienna, Austria) according to the manufacturer’s instructions. The products of the sequencing reaction were separated on an ABI Prism 3130xl Genetic Analyzer. Analysis was performed with SeqScape v2.5. All amplicons (details see Table S3) were sequenced in both directions. All tumor samples were analyzed for TP53 mutations by sequencing exons 4–9. Those tumors which were p53 IHC positive but exhibited no mutation in exons 4–9 (n = 8) were additionally analyzed for mutations in exons 10–11. The sequencing primers and the complete list of TP53 mutations found in our cohort are shown in Table S3 and S4, respectively. The copy numbers of TP53 were determined in tumor samples using a TaqMan copy number assay (hs06423639_cn, Applied Biosystems), which was analyzed simultaneously with a reference assay (RNaseP; Cat#4403326, Applied Biosystems) in a duplex qPCR. The CopyCaller Software from Applied Biosystems was used for post-PCR quantitative analysis of copy numbers.

**Table 3:** Association of the TP53 Arg72Propolymorphism with breast cancer risk in the indicated subgroups.

	Category	No. of cases (%)	Pro/Pro vs. Arg/Arg		Arg/Pro vs. Arg/Arg		Pro vs. Arg	
			OR	95% CI	OR	95% CI	OR	95% CI
Age (years)^1^	<55	106 (39.7%)	2.10	(0.73–6.10)	1.30	(0.80–2.10)	1.36	(0.92–2.02)
	≥55	161 (60.3%)	2.57	(1.00–6.58)	1.50	(0.98–2.28)	1.54	(1.09–2.18)*
Tumor type	ductal	148 (75.9%)	1.90	(0.70–5.14)	1.26	(0.82–1.94)	1.31	(0.92–1.87)
	lobular	47 (24.1%)	4.34	(1.28–14.7)*	1.92	(0.98–3.74)	2.01	(1.20–3.37)**
Grade	pG1-2	157 (64.1%)	3.08	(1.21–7.80)*	1.70	(1.11–2.60)*	1.72	(1.22–2.44)**
	pG3	88 (35.9%)	1.59	(0.50–5.11)	1.00	(0.60–1.67)	1.10	(0.72–1.68)
p53 status	pos	56 (22.7%)	1.62	(0.40–6.47)	1.19	(0.65–2.18)	1.22	(0.74–2.02)
	neg	191 (77.3%)	2.76	(1.12–6.79)*	1.54	(1.03–2.30)*	1.59	(1.14–2.21)**
ER status	pos	196 (77.2%)	2.73	(1.11–6.71)*	1.59	(1.06–2.37)*	1.61	(1.16–2.24)**
	neg	58 (22.8%)	2.08	(0.59–7.38)	1.15	(0.63–2.10)	1.27	(0.78–2.07)
PR status	pos	137 (53.9%)	3.07	(1.17–8.05)*	1.80	(1.15–2.08)*	1.78	(1.24–2.55)**
	neg	117 (46.1%)	2.08	(0.75–5.82)	1.17	(0.74–1.87)	1.28	(0.88–1.88)
HER2 status	pos	51 (20.3%)	2.60	(0.80–8.53)	0.77	(0.39–1.49)	1.13	(0.68–1.88)
	neg	200 (79.7%)	2.57	(1.03–6.40)*	1.71	(1.15–2.54)**	1.66	(1.20–2.31)**

ER, estrogen receptor; PR, progesterone receptor; 95% CI, 95% confidence intervals.

1 patients aged under 55 years or ≥55 years at diagnosis were compared to control subjects of any age for sake of comparability with the other subgroup analyses.

* indicates p-values <0.05; **indicates p-values <0.01.

### qRT-PCR Analysis of mRNA Expression

RNA extraction, cDNA synthesis, and qRT-PCR were described in detail previously [46]. Briefly, total RNA was isolated with TRIreagent (Sigma), quality-controlled with the Bioanalyzer 2100 (Agilent), and reverse-transcribed with the high-capacity cDNA archive Kit (Applied Biosystems) according to the manufacturers’ instructions. Each sample was analyzed in duplicate by a real-time PCR on an Applied Biosystems 7500 fast instrument, using gene-specific primers and fluorescent probes obtained from Applied Biosystems: p53, hs_001533340_m1; MDM2, hs00234753_m1; p21, hs00355782_m1; BAX, hs00414514_m1; PERP, hs00953482_g1; and β-actin (control), hs_99999903_m1. The mRNAs levels of p53, MDM2, p21, BAX and PERP were normalized to those of β-actin in each sample by subtracting the Ct (threshold cycle) value of β-actin from the Ct value of each of these genes. This subtraction produces ΔCt (deltaCt)-values, e.g. ΔCt_p53_ =  Ct_p53_ - Ct_β-actin_, which are shown in Table S2. Relative mRNA expression levels were derived from ΔCt-values as 2^-ΔCt^. These relative mRNA levels were further normalized to each other, thus deriving 2^-ΔΔCt^ values, as follows: For unselected patients, the levels of Arg/Arg patients were set to unity (1), and the levels of Pro-carriers were expressed relative to those of Arg/Arg patients. For patients stratified by TP53 status, the levels of Arg/Arg patients with wildtype p53 were set to unity, and the levels of Arg/Arg patients with mutant p53, and of Pro-carriers with wildtype or mutant p53 were expressed relative to those. Thus, the impact of both the Arg72Pro genotype and p53 status upon expression of p53, MDM2, p21, BAX and PERP is revealed. mRNA expression analyses are based on those 73 samples with complete data, i.e. in which mRNA levels of all 5 genes, the Arg72Pro genotype, as well as the p53 status by sequencing and immuno histochemistry could be successfully determined.

**Figure 2:**
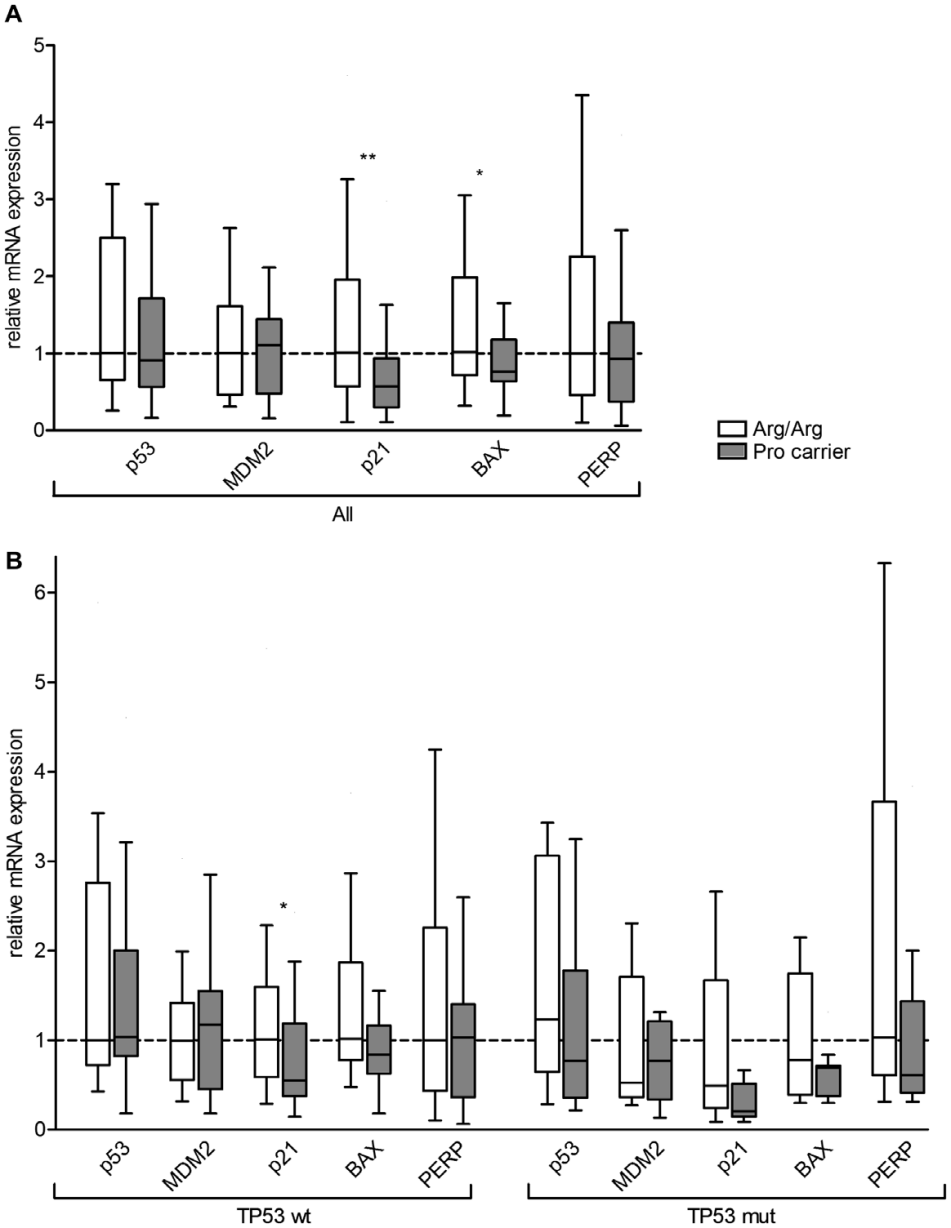
Expression of p53 target genes in human breast tumors. Boxplots of the intratumoral p53, MDM2, p21, BAX and PERP mRNA levels of breast cancer patients, normalized to β-actin. **A,** Results for unselected patients with genotype Arg/Arg (n = 36) and Pro-allele carriers (n = 37). Median mRNA expression levels of Arg/Arg patients were set to 1, and median levels of Pro-allele carriers (Arg/Pro, Pro/Pro) are expressed relative to the levels of Arg/Arg patients. **B,** Results for patients with TP53 wildtype (wt) tumors with genotype Arg/Arg (n = 24) and for Pro-allele carriers (n = 25), and patients with TP53 mutated (mut) tumors with genotype Arg/Arg (n = 12) and for Pro-allele carriers (n = 12). Median mRNA expression levels of Arg/Arg patients with wildtype p53 were set to unity, and the levels of Arg/Arg patients with mutant p53, and of Pro-carriers with wildtype or mutant p53 are expressed relative to those. **indicates p-values <0.01; *indicates p-values <0.05 (Wilcoxon-Mann-Whitney U-Test).

### Survival Analysis

All survival analyses were based on the population of 118 breast cancer patients from whom fresh-frozen tumor tissue was collected between 1991 and 1994, since detailed and long-term follow-up data have been documented for this population. Two patients were omitted from these analyses due to missing genotypes, and one due to missing follow-up data. The mean follow-up times of the remaining 115 patients were 6.9±5.3 years (median, 5.5 years; range, 0–18.3 years) for the overall survival, and 5.3±5.2 years (median, 3.2 years; range, 0–14.5 years) for the disease-free survival. For those patients who were still alive or disease-free, respectively, at the end of follow up, the mean follow-up times were 10.0±5.8 years (median, 12.8 years; range, 0–18.3 years) for the overall survival, and 9.0±5.6 years (median, 12.5 years; range, 0–14.5 years) for the disease-free survival. All Kaplan-Meier analyses were truncated to 10 years. Accordingly, all patients with follow-up times >10.1 years were censored at 10.1 years for these analyses. Patients who were lost from follow-up before that time and were event-free were also censored. The number of events in this 10-year period was 59 for the overall survival (42 in patients with p53 wildtype tumors, 17 in p53 mutant patients), and 65 for the disease-free survival (43 in p53 wildtype, 22 in p53 mutant patients). After these ten years, another 8 events for the overall survival and 3 events for the disease-free survival had occurred. “Event” was defined as breast-cancer related death in the overall survival, and as affirmation of a distant metastasis, a second primary breast tumor, or a recurrent primary tumor in the disease-free survival. In the curves of cumulative breast cancer incidence in Figure 1D–F, there were no censored data since the age at breast cancer onset was known for each patient. Likewise, ages at interview were known for all controls. Overall and disease-free survival were also analyzed by using a Cox proportional hazard model, unadjusted or adjusted for TP53 mutation status, ER status, HER2 status, and grading. Cox proportional hazard models and Kaplan-Meier plots were computed with the R survival package [47].

### Additional Statistical Analysis

Statistical analyses were performed with SPSS 17.0 and R, an open-source language and environment for statistical computing [47]. Chi-square tests with Yates’ continuity correction were used to evaluate potential deviations of the study population from Hardy-Weinberg equilibrium. Confidence intervals given are 95% mid-P exact confidence intervals. The p-values shown in Table 2 are mid-P two-tailed exact p-values. We consider the results of our subgroup analyses in Table 3 as exploratory, and hence did not adjust for multiple testing, as recommended previously [48]. Differences between the three Arg72Pro genotypes with respect to age of breast cancer onset were analyzed as described [49]. mRNA levels of p53, MDM2, p21, BAX and PERP were analyzed by Wilcoxon-Mann-Whitney U-Test. P<0.05 was considered significant.

**Figure 3:**
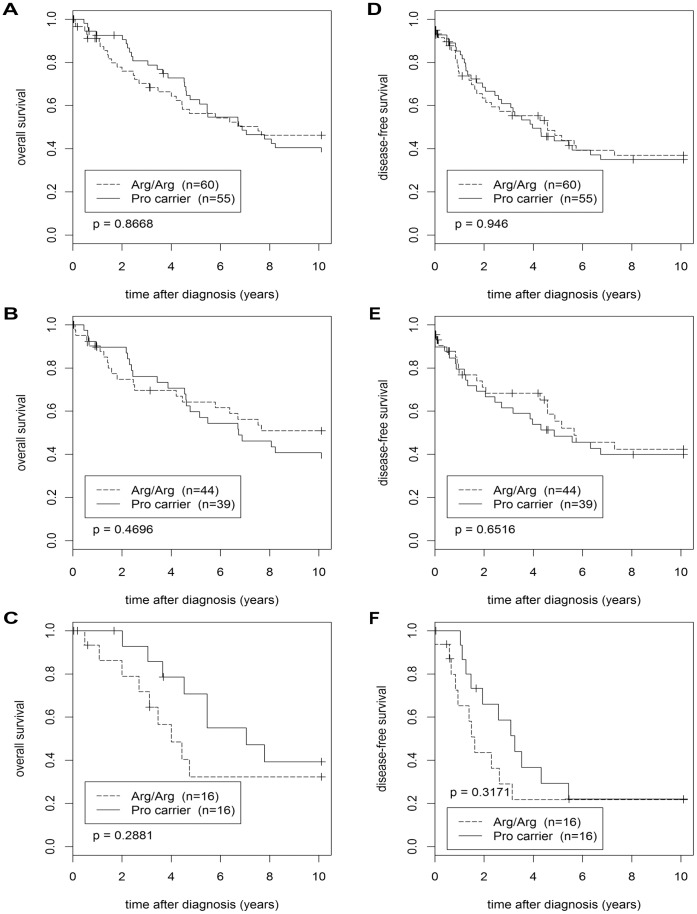
Kaplan-Meier analyses of the overall and disease-free survival. Overall survival (**A–C**) and disease-free survival (**D–F**) of patients with the Arg/Arg genotype and of Pro-allele carriers was compared. Kaplan-Meier analyses for unselected patients (**A, D**), patients with TP53 wildtype tumors (**B, E**), and patients with mutated TP53 in their tumors (**C, F**) are shown.

**Table 4:** Univariable and multivariable analyses of the overall survival using a Cox proportional hazards model.

		Univariable		Multivariable	
Variable	Subcategory	Hazard Ratio	p-value	Hazard Ratio	p-value
SNP genotype	Arg/Arg = 0, Pro-carrier = 1	0.97 (0.60–1.57)	0.8990	1.14 (0.65–1.98)	0.6495
TP53 status	wt = 0, mutated = 1	1.13 (0.66–1.94)	0.6630	0.75 (0.39–1.43)	0.3766
ER status	pos = 0, neg = 1	1.10 (0.63–1.90)	0.7410	1.16 (0.66–2.04)	0.6007
HER2 status	neg = 0, pos = 1	2.60 (1.39–4.84)	0.0027	2.71 (1.43–5.13)	0.0023
Grading	pG1-2 = 0, pG3 = 1	1.71 (1.06–2.78)	0.0292	1.62 (0.91–2.87)	0.1018

**Table 5:** Univariable and multivariable analyses of the disease-free survival using a Cox proportional hazards model.

	Univariable		Multivariable	
Variable*	Hazard Ratio	p-value	Hazard Ratio	p-value
SNP genotype	0.96 (0.59–1.54)	0.8570	1.03 (0.58–1.83)	0.9136
TP53 status	1.66 (0.99–2.78)	0.0533	1.23 (0.65–2.33)	0.5277
ER status	1.60 (0.90–2.87)	0.1120	1.65 (0.90–3.02)	0.1074
HER2 status	2.69 (1.44–5.03)	0.0020	2.83 (1.46–5.47)	0.0021
Grading	1.45 (0.89–2.36)	0.1400	1.04 (0.58–1.87)	0.8955

* Subcategories of the indicated variables were coded as in Table 4.

## Results

### TP53 Arg72Pro SNP and Breast Cancer Risk

A single nucleotide polymorphism (SNP) in codon 72 of the TP53 gene (Arg72Pro) was genotyped in 487 individuals (267 consecutive breast cancer patients and 220 female control subjects). Clinical characteristics of the study population, and the frequency of the Arg72Pro genotypes in the study population and subpopulations thereof are shown in Table 1. Both the control population and the breast cancer patients were in Hardy-Weinberg equilibrium (p = 0.17 and p = 0.16, respectively). The frequency of the minor Pro-allele was 30.1% in patients and 23.4% in controls. To determine odds ratios and 95% confidence intervals for breast cancer risk, various comparisons of TP53 Arg72Pro genotypes as well as Pro vs. Arg alleles were analyzed. We found markedly increased odds ratios associated with the presence of one or two Pro-alleles, three of which were statistically significant: Pro/Pro vs. Arg/Arg (OR, 2.38; 95% CI, 1.01–5.93; p = 0.046), Pro/Pro+Arg/Pro vs. Arg/Arg (OR, 1.49; 95% CI, 1.04–2.14; p = 0.028), and Pro vs. Arg (OR, 1.46; 95% CI, 1.08–1.99; p = 0.013; Table 2). The result of the latter analysis (Pro vs. Arg) remained significant when corrected odds ratios adjusted for age and menopausal status were calculated (OR, 1.60; 95% CI, 1.02–2.50; p = 0.036; Table 2).

Next, odds ratios associated with the Pro-allele were evaluated in specific breast cancer subpopulations, and were found considerably elevated in several patient subgroups (Table 3). In this exploratory analysis, higher odds ratios were determined in patients with a lobular tumor type, and in patients with a low-grade tumor (pG1 or 2 vs. pG3). Moreover, odds ratios associated with the Pro-allele were significantly elevated in ER positive, PR positive, and in HER2 negative patients (Table 3). Importantly, the impact of the Arg72Pro SNP on breast cancer risk appears to be limited to p53 negative patients, whereas odds ratios did not significantly deviate from unity in patients with p53 positive tumors, in which p53 is inactivated by mutation (Table 3).

We next analyzed the impact of the Arg72Pro genotype upon the age of breast cancer onset. Interestingly, patients with the Pro/Pro genotype were diagnosed with breast cancer at a mean age of 63.2±17.9 years (median, 67.6), whereas Arg/Arg patients were diagnosed at 58.2±15.0 years (median, 60.2), and Arg/Pro patients at 58.5±13.0 years (median, 58.1; Figure 1A). Thus, Pro/Pro patients were diagnosed with breast cancer significantly later than patients with the other two genotypes (p = 0.0497; Figure 1A, D). This difference was even more pronounced in p53 negative patients (p = 0.0308; Figure 1B, E). In this subpopulation, Pro/Pro patients were diagnosed at 65.8±16.7 years (median, 69.7), heterozygous patients at 59.9±12.7 years (median, 62.0), and Arg/Arg patients at 61.5±14.5 years (median, 62.5; Figure 1B, E). In contrast, this effect was not observed in p53 positive patients (Figure 1C, F).

### TP53 Arg72Pro SNP and mRNA Levels of p53, MDM2, p21, BAX, and PERP

The Arg72Pro SNP was also genotyped in 116 fresh frozen breast tumor tissue samples. The frequency of the Pro-allele was 25.9% in these patients. We next analyzed the impact of the Arg72Pro genotype upon the mRNA expression of p53 itself and its key target genes MDM2, p21, BAX and PERP in these tumor samples (Figure 2). As the number of Pro/Pro patients was small, we compared homozygous and heterozygous carriers of the Pro-allele (genotypes Pro/Pro+Arg/Pro; n = 37) to homozygous carriers of the Arg-allele (genotype Arg/Arg; n = 36). However, raw expression values of all three genotypes are shown in Table S2. In patients unselected for p53 mutation status, the mRNA levels of p53, MDM2, and PERP were only marginally affected by Arg72Pro genotypes (Figure 2A). In contrast, the median mRNA level of p21 was significantly reduced to 56% (p = 0.008, Wilcoxon-Mann-Whitney U-Test), and BAX mRNA expression was reduced to 77% (p = 0.041) in the tumors of Pro-allele carriers compared to those of Arg/Arg patients (Figure 2A). Interestingly, a significant reduction of the transcript levels of these p53 target genes in Pro-allele carriers was not observed in TP53-mutated tumors (Figure 2B). In contrast, in patients with wildtype TP53 the median mRNA level of p21 was significantly reduced to 54% (p = 0.038) in Pro-allele carriers compared to Arg/Arg patients (Figure 2B), whereas the levels of BAX were reduced non-significantly (p = 0.114; Figure 2B). Interestingly, expression of MDM2 tended to be slightly elevated in Pro-allele carriers, although these differences were not significant (Figure 2). Expression of PERP was hardly influenced both by Arg72Pro genotype and by mutational inactivation of TP53, whereas expression levels of the other three p53 target genes were considerably lower in tumors with mutated TP53 (Figure 2B). Expression of p53 and its target genes could also be affected by variations in copy number of the TP53 gene in tumors. Accordingly, we performed copy number analysis of TP53 with tumor-derived DNA of our study population. One patient had only one copy of the TP53 gene, who had also a missense mutation in codon 127 (Table S4); all others had two copies. This patient with one copy of TP53 was not included in the mRNA expression analyses.

### TP53 Arg72Pro SNP and Prognosis

We next performed Kaplan-Meier analyses of the overall and disease-free survival, comparing Pro-allele carriers with patients with the homozygous Arg/Arg genotype (Figure 3). We grouped Pro/Pro patients (n = 4; 1 with mutated TP53 in her tumor, 3 with wildtype p53) together with Arg/Pro patients since their number was too small for a separate analysis. In these four Pro/Pro patients, 2 events were observed in the analysis of overall survival (one each in patients with wildtype and mutant p53), and 3 events in the disease-free survival (2 of them in patients with wildtype p53). These Kaplan-Meier analyses were performed in the entire population (n = 115), and separately in patients with wildtype TP53 (n = 83) and in patients with mutant TP53 in their tumors (n = 32). No significant differences in the survival of Pro carriers vs. Arg/Arg patients were observed in any of the six analyses (Figure 3). Pro carriers tended to have a slightly poorer overall survival than Arg/Arg patients in the wildtype p53 sub-population, however, this difference was not significant (Figure 3B). We next performed multivariable analysis of the overall and disease-free survival using a Cox proportional hazards model adjusted for TP53-, ER-, and HER2-status as well as grading (Table 4 and 5). In a parallel univariable analysis, each variable was also analyzed individually. HER2-status was the strongest independent prognostic factor in these analyses, whereas no significant differences in the survival of Pro carriers vs. Arg/Arg patients were observed (Table 4 and 5).

## Discussion

The Arg72Pro SNP affects the amino acid sequence of p53, and different biochemical properties have been reported for the two resulting p53 variants [21]–[23], [26], [27]. p53^72Arg^ is more efficient in inducing apoptosis, whereas p53^72Pro^ exhibits higher DNA-repair capacity and is a stronger inducer of cell cycle arrest [8], [21]–[23], [26], [27]. Our findings are consistent with a model in which the p53^72Arg^ variant is a more potent tumor suppressor than p53^72Pro^, presumably mainly due to inducing target genes with a key role in apoptosis and cell cycle arrest more efficiently. Consistent with this model, the supposably weaker tumor suppressor p53^72Pro^ was associated with an increased breast cancer risk (Table 2). An increased breast cancer risk associated with the Pro-allele has also been found by several previous studies [28], [29], [50]. However, most of the larger studies and meta-analyses did not find the Arg72Pro SNP to be associated with breast cancer risk [31], [33], [35]–[41]. This SNP exhibits pronounced differences in allele frequencies as a function of geographical latitude, the Arg-allele becoming progressively more frequent the further North a population resides, and it has been suggested that this may confound association studies if the study population is recruited from more than one geographical area [42], [43].

We also observed a ∼5 year older mean age at breast cancer onset in Pro/Pro patients compared to Arg/Pro and Arg/Arg patients (Figure 1). This is consistent with a study of Li-Fraumeni patients, in which the mean age of tumor onset in Pro/Pro patients was 34.4 years, and of Arg carriers 21.8 years [51]. Conceivably, more severe somatic mutations are required to permit tumor progression in the presence of the p53^72Arg^ variant, which we propose to be the stronger tumor suppressor (see above). These more severe mutations could also accelerate tumor progression and hence shorten the time period from tumor initiation to when the tumor becomes clinically manifest, thus leading to a younger age at onset. One obvious candidate for such a somatic mutation is inactivation of p53 itself, which is indeed more frequent in carriers of the p53^Arg72^ variant: 25.4% p53 positive tumors were found in Arg/Arg patients (29/114), 20.9% in Arg/Pro (24/115), and 16.7% in Pro/Pro (3/18; Table 1). This is consistent with a previous report, in which TP53 was found mutated in 28.5% of Arg/Arg patients, but only 3.8% of Pro/Pro patients [52], and may reflect a higher selective pressure to mutate the supposably more potent tumor suppressor p53^72Arg^. Moreover, a larger fraction of the tumors of Arg/Arg patients exhibited markers of high malignancy and aggressiveness, such as a high grade, ER and PR negativity, and HER2 positivity, which may also indicate that more severe mutations had occurred in these Arg/Arg tumors (Table 1 and 3). On the other hand, Pro/Pro patients with p53 negative tumors were reported to have a poorer breast cancer-specific survival than Arg/Arg or Arg/Pro patients [35], [44]. In our analysis of Pro-carriers vs. Arg/Arg patients, we found no significant survival differences, although Pro-carriers with p53 negative tumors tended to have a slightly poorer long-term overall survival than p53 negative Arg/Arg patients (Figure 3B).

The issue whether one of the two p53 variants is a more efficient transcriptional activator than the other is still controversial and relies mostly on *in vitro* studies. The relative transcriptional activities of p53^72Arg^ and p53^72Pro^ remain to be clarified, and seem to depend on the experimental system used and target genes analyzed [8], [22], [23], [26], [27]. In the most comprehensive *in vitro* study, 34 p53 target genes were analyzed in Saos-2 osteosarcoma cells [8]. In this system, PERP was induced ∼1000-fold by p53^72Arg^, but only ∼12-fold by p53^72Pro^. 10 other genes were also induced >2-fold better by p53^72Arg^, but none by p53^72Pro^. The only *in vivo* study so far has analyzed the peripheral leukocytes, but not the tumors, of human lung cancer patients and controls, in which Pro-allele carriers exhibited 38% lower p21 mRNA levels than corresponding subjects with the Arg/Arg genotype, which agrees well with our results reported here [25]. To the best of our knowledge, we report the first analysis of the association of TP53 Arg72Pro genotypes with p53, MDM2, p21, BAX and PERP mRNA expression in human breast tumor specimen. In contrast to a previous report [8], we did not observe any significant effect of the Arg72 Pro genotype on PERP-expression, which is likely due to the fact that we analyzed steady-state rather than induced mRNA levels. However, we found significantly reduced p21 and BAX mRNA expression levels in the tumors of Pro-allele carriers.

In conclusion, we could show a significantly enhanced breast cancer risk associated with the Pro-allele, a significantly later age at breast cancer onset for Pro/Pro patients, and significantly lower p21 and BAX mRNA levels in the tumors of Pro-allele carriers. However, none of these effects were observed in patients with somatic TP53 mutations in their tumors. It is biologically plausible that any biochemical differences that may exist between p53^72Arg^ and p53^72Pro^ become irrelevant in the event of functional inactivation of p53. Collectively, our data indicate that the p53^72Arg^ variant is a more potent *in vivo* transcriptional activator and tumor suppressor in human breast cancer patients than the p53^72Pro^ variant.

## Supporting Information

Table S1 **Clinical characteristics of the study population (tumor tissue cohort), and frequency of the p53^Arg72Pro^ genotypes in the indicated subpopulations.**
(DOCX)

Table S2 **Raw mRNA expression levels (ΔCt values) of p53 and its target genes in the indicated subgroups stratified by TP53 status and Arg72Pro genotype.**
(DOCX)

Table S3 **Primers used for TP53 sequencing.**
(DOCX)

Table S4 **Overview of TP53 mutations found in our study population.**
(DOCX)
