# Supplementary material for: The Pro Allele of the p53 Codon 72 Polymorphism Is Associated with Decreased Intratumoral Expression of BAX and p21, and Increased Breast Cancer Risk
Source: PLoS One. 2012 Oct 10;7(10):e47325. doi: 10.1371/journal.pone.0047325 (PMC3468577; doi:10.1371/journal.pone.0047325)
Supplement: Table S1 — Clinical characteristics of the study population (tumor tissue cohort), and frequency of the p53Arg72Pro genotypes in the indicated subpopulations. (DOCX) [file pone.0047325.s001.docx]

**Table S1.** Clinical characteristics of the study population (tumor tissue cohort), and frequency of the p53^Arg72Pro^ genotypes in the indicated subpopulations.

|  |  | Total | Arg/Arg | Arg/Pro | Pro/Pro |
| --- | --- | --- | --- | --- | --- |
|  | | (n=115) | (n=59) | (n=52) | (n=4) |
| Mean Age (years) | | 55.9±12.6 | 56.7±12.8 | 55.1±12.4 | 56.4±14.4 |
| Median Age (years) | | 56.1 | 54.9 | 56.4 | 58.2 |
| menopausal | prae | 26 | 13 (50,0%) | 13 (50,0%) | 0 (0,0%) |
| status | post | 51 | 21 (41,2%) | 28 (54,9%) | 2 (3,9%) |
|  | na | 38 | 25 (65,8%) | 11 (28,9%) | 2 (5,3%) |
| Tumorsize | pT1 | 34 | 15 (44.1%) | 18 (52.9%) | 1 (2.9%) |
|  | pT2 | 65 | 35 (53.8%) | 28 (43.1%) | 2 (3.1%) |
|  | pT3, pT4 | 13 | 8 (61.5%) | 4 (30.8%) | 1 (7.7%) |
|  | other, na | 3 | 1 (33.3%) | 2 (66.7%) | 0 (0.0%) |
| Tumortype | ductal | 89 | 46 (51.7%) | 40 (44.9%) | 3 (3.4%) |
|  | lobular | 21 | 10 (47.6%) | 10 (47.6%) | 1 (4.8%) |
|  | other, na | 5 | 3 (60.0%) | 2 (40.0%) | 0 (0.0%) |
| Stage | 0, I | 22 | 9 (40.9%) | 13 (59.1%) | 0 (0.0%) |
|  | II | 65 | 37 (56.9%) | 26 (40.0%) | 2 (3.1%) |
|  | III, IV | 16 | 9 (56.3%) | 6 (37.5%) | 1 (6.3%) |
|  | na | 12 | 4 (33.3%) | 7 (58.3%) | 1 (8.3%) |
| Grade | pG1 | 3 | 1 (33.3%) | 2 (66.7%) | 0 (0.0%) |
|  | pG2 | 59 | 30 (50.8%) | 28 (47.5%) | 1 (1.7%) |
|  | pG3 | 50 | 27 (54.0%) | 21 (42.0%) | 2 (4.0%) |
|  | na | 3 | 1 (33.3%) | 1 (33.3%) | 1 (33.3%) |
| Nodal status | pN0 | 40 | 17 (42.5%) | 21 (52.5%) | 2 (5.0%) |
|  | pN+ | 70 | 39 (55.7%) | 29 (41.4%) | 2 (2.9%) |
|  | na | 5 | 3 (60.0%) | 2 (40.0%) | 0 (0.0%) |
| TP53 status | wild-type | 83 | 43 (51.8%) | 37 (44.6%) | 3 (3.6%) |
|  | mutated | 32 | 16 (50.0%) | 15 (46.9%) | 1 (3.1%) |
| ER status | neg | 55 | 27 (49.1%) | 27 (49.1%) | 1 (1.8%) |
|  | pos | 34 | 16 (47.1%) | 18 (52.9%) | 0 (0.0%) |
|  | na | 26 | 16 (61.5%) | 7 (26.9%) | 3 (11.5%) |
| PR status | neg | 60 | 28 (46.7%) | 30 (50.0%) | 2 (3.3%) |
|  | pos | 24 | 12 (50.0%) | 12 (50.0%) | 0 (0.0%) |
|  | na | 31 | 19 (61.3%) | 10 (32.3%) | 2 (6.5%) |
| HER2 status | neg | 72 | 32 (44.4%) | 39 (54.2%) | 1 (1.4%) |
|  | pos | 18 | 10 (55.6%) | 7 (38.9%) | 1 (5.6%) |
|  | na | 25 | 17 (68.0%) | 6 (24.0%) | 2 (8.0%) |
