# Supplementary material for: The Pro Allele of the p53 Codon 72 Polymorphism Is Associated with Decreased Intratumoral Expression of BAX and p21, and Increased Breast Cancer Risk
Source: PLoS One. 2012 Oct 10;7(10):e47325. doi: 10.1371/journal.pone.0047325 (PMC3468577; doi:10.1371/journal.pone.0047325)
Supplement: Table S2 — Raw mRNA expression levels (ΔCt values) of p53 and its target genes in the indicated subgroups stratified by TP53 status and Arg72Pro genotype. (DOCX) [file pone.0047325.s002.docx]

**Table S2.** Raw mRNA expression levels (ΔCt values) of p53 and its target genes in the indicated subgroups stratified by TP53 status and Arg72Pro genotype.

| TP53 status | | All | | | TP53 wt | | | TP53 mut | | |
| --- | --- | --- | --- | --- | --- | --- | --- | --- | --- | --- |
| Arg72Pro genotype | | Arg/Arg | Arg/Pro | Pro/Pro | Arg/Arg | Arg/Pro | Pro/Pro | Arg/Arg | Arg/Pro | Pro/Pro |
|  |  | (n=36) | (n=37) | (n=1) | (n=24) | (n=25) | (n=0) | (n=12) | (n=11) | (n=1) |
| ΔCt p53 | Percentile 25 | 3,73 | 4,30 |  | 3,73 | 4,18 |  | 3,58 | 4,53 |  |
|  | Median | 4,90 | 5,15 | 4,03 | 5,24 | 5,11 |  | 4,64 | 5,78 | 4,03 |
|  | Percentile 75 | 5,62 | 5,84 |  | 5,62 | 5,42 |  | 5,95 | 6,83 |  |
| ΔCt MDM2 | Percentile 25 | 4,95 | 5,13 |  | 4,95 | 4,86 |  | 4,78 | 5,28 |  |
|  | Median | 5,69 | 5,55 | 5,17 | 5,50 | 5,27 |  | 6,55 | 6,04 | 5,17 |
|  | Percentile 75 | 6,80 | 6,75 |  | 6,35 | 6,67 |  | 6,97 | 7,10 |  |
| ΔCt p21 | Percentile 25 | 4,38 | 5,19 |  | 4,38 | 4,60 |  | 4,47 | 6,49 |  |
|  | Median | 5,24 | 6,00 | 6,84 | 4,73 | 5,84 |  | 5,93 | 7,32 | 6,84 |
|  | Percentile 75 | 5,97 | 6,86 |  | 5,65 | 6,33 |  | 6,95 | 7,61 |  |
| ΔCt BAX | Percentile 25 | 5,02 | 5,76 |  | 5,02 | 5,71 |  | 5,15 | 6,37 |  |
|  | Median | 5,91 | 6,37 | 6,35 | 5,90 | 6,30 |  | 6,28 | 6,54 | 6,35 |
|  | Percentile 75 | 6,50 | 6,64 |  | 6,26 | 6,59 |  | 7,43 | 7,23 |  |
| ΔCt PERP | Percentile 25 | 3,20 | 3,94 |  | 3,20 | 3,88 |  | 2,61 | 4,25 |  |
|  | Median | 4,37 | 4,51 | 3,73 | 4,37 | 4,32 |  | 4,33 | 5,13 | 3,73 |
|  | Percentile 75 | 5,48 | 5,83 |  | 5,57 | 5,85 |  | 5,08 | 5,83 |  |
