# Supplementary material for: The Pro Allele of the p53 Codon 72 Polymorphism Is Associated with Decreased Intratumoral Expression of BAX and p21, and Increased Breast Cancer Risk
Source: PLoS One. 2012 Oct 10;7(10):e47325. doi: 10.1371/journal.pone.0047325 (PMC3468577; doi:10.1371/journal.pone.0047325)
Supplement: Table S3 — Primers used for TP53 sequencing. (DOCX) [file pone.0047325.s003.docx]

**Table S3.** Primers used for TP53 sequencing.

| Fragment | Sequence | Size* |
| --- | --- | --- |
| Exon4 | F: 5´- CTC TGA CTG CTC TTT TCA CC -3´ |  |
|  | R: 5´- CAT TGA AGT CTC ATG GAA GC -3´ | 348bp |
| Exon5-6 | F: 5´- GTT TCT TTG CTG CCG TCT TC -3´ |  |
|  | R: 5´- TAA CCC CTC CTC CCA GAG AC -3´ | 498bp |
| Exon7-9 | F: 5´- AAG GCG CAC TGG CCT CAT CTT -3´ |  |
|  | R: 5´- TCC ACT TGA TAA GAG GTC CC -3´ | 828bp |
| Exon10 | F: 5´- CAA TTG TAA CTT GAA CCA TC -3´ |  |
|  | R: 5´- GAT GAG AAT GGA ATC CTA TG -3´ | 259bp |
| Exon11 | F: 5´- ATC TCT CCT CCC TGC TTC TG -3´ |  |
|  | R: 5´- AGG CTG TCA GTG GGG AAC AA -3´ | 145bp |

*Size of the amplicon generated with the indicated primer pairs. annealing temperature was 55°C for all primer sets
