# Supplementary material for: The Pro Allele of the p53 Codon 72 Polymorphism Is Associated with Decreased Intratumoral Expression of BAX and p21, and Increased Breast Cancer Risk
Source: PLoS One. 2012 Oct 10;7(10):e47325. doi: 10.1371/journal.pone.0047325 (PMC3468577; doi:10.1371/journal.pone.0047325)
Supplement: Table S4 — Overview of TP53 mutations found in our study population. (DOCX) [file pone.0047325.s004.docx]

**Table S4.** Overview of TP53 mutations found in our study population.

| ID | Genotype (Codon72) | Exon | Codon | Codon change | Base change | Amino acid change | Type |
| --- | --- | --- | --- | --- | --- | --- | --- |
| 5* | Arg/Arg | 5 | 127 | TCC > TTC | C>T | Ser > Phe | missense |
| 11 | Pro/Pro | 7 | 242 | TGC > TTC | G>T | Cys > Phe | missense |
| 12 | Arg/Pro | 4 | 108 | 1 bp del |  |  | frameshift |
| 15 | Arg/Arg | 7 | 237 | ATG > ATA | G>A | Met > Ile | missense |
| 18 | Arg/Arg | 5 | 179 | CAT > TAT | C>T | His > Tyr | missense |
| 22 | Arg/Arg | 6 | 193 | CAT > CCT | A>C | His > Pro | missense |
| 24 | Arg/Pro | 8 | 275 | TGT > TAT | G>A | Cys > Tyr | missense |
| 25 | Arg/Arg | 4 | 107 | TAC > TAG | C>G | Tyr > Stop | nonsense |
| 29 | Arg/Arg | 10 | 346 | GAG > TAG | G>T | Glu > Stop | nonsense |
| 32 | Arg/Arg | 7 | 245 | GGC > GCC | G>C | Gly > Ala | missense |
| 33 | Arg/Pro | 7 | 241 | 10 bp ins |  |  | frameshift |
| 36 | Arg/Arg | 7 | 231 | 7 bp del |  |  | frameshift |
| 37 | Arg/Pro | 5 | 175 | CGC > CAC | G>A | Arg > His | missense |
| 40 | Arg/Arg | 5 | 173 | GTG > ATG | G>A | Val > Met | missense |
| 45 | Arg/Arg | 4 | 113 | TTC > TCC | T>C | Phe > Ser | missense |
| 47 | Arg/Pro | 6 | 220 | TAT > TGT | A>G | Tyr > Cys | missense |
| 51 | Arg/Pro | 7 | 251 | 1 bp del |  |  | frameshift |
| 53 | Arg/Arg | 8 | 280 | AGA > ATA | G>T | Arg > Ile | missense |
| 56 | Arg/Pro | 6 | 204 | GAG > TAG | G>T | Glu > Stop | nonsense |
| 58 | Arg/Arg | 7 | 242 | TGC > TCC | G>C | Cys > Ser | missense |
| 59 | Arg/Pro | 4 | 110 | 6 bp ins |  |  | in frame |
| 63 | Arg/Pro | 5 | 175 | CGC > CAC | G>A | Arg > His | missense |
| 68 | Arg/Arg | 6 | 192 | CAG > TAG | C>T | Gln > Stop | nonsense |
| 71 | Arg/Pro | 8 | 267 | 10 bp del |  |  | frameshift |
| 75 | Arg/Arg | 5 | 176 | TGC > TTC | G>T | Cys > Phe | missense |
| 77 | Arg/Pro | 8 | 273 | CGT > TGT | C>T | Arg > Cys | missense |
| 82 | Arg/Pro | 8 | 278 | CCT > GCT | C>G | Pro > Ala | missense |
| 86 | Arg/Arg | 8 | 282 | CGG > CCG | G>C | Arg > Pro | missense |
| 91 | Arg/Pro | 7 | 229 | 2 bp del |  |  | frameshift |
| 97 | Arg/Arg | 7 | 245 | GGC > TGC | G>T | Gly > Cys | missense |
| 101 | Arg/Pro | 7 | 248 | CGG > GGG | C>G | Arg > Gly | missense |
| 110 | Arg/Pro | I6 | c_672+1 | Intronic | G>T |  | splice |

Most mutations are already listed in the IARC TP53 database [20]. *this tumor has only one copy of the TP53 gene.
